# Characterizing the within-person variability of food insecurity in everyday life

**DOI:** 10.1371/journal.pone.0312543

**Published:** 2025-01-17

**Authors:** Caitlin T. Hines, Rebecca M. Ryan, Joshua M. Smyth

**Affiliations:** 1 Department of Biobehavioral Health, The Pennsylvania State University, University Park, Pennsylvania, United States of America; 2 Department of Psychology, Georgetown University, Washington, DC, United States of America; 3 Department of Psychology, The Ohio State University, Columbus, Ohio, United States of America; Sindh Social Protection Authority, PAKISTAN

## Abstract

Food insecurity (FI), the lack of access to adequate food, is linked with negative health and psychological outcomes. FI is typically measured retrospectively over the last year; although this measurement is useful to understand FI prevalence to inform broad policy, it leaves the experience of FI in everyday life poorly understood. Understanding how FI varies across shorter periods of time (days or weeks) can help inform FI prevention and/or intervention. This study characterizes within-person (day-to-day) variance in FI in everyday life. Low-income parents of school-aged children at risk for FI (*n* = 153) completed daily text message surveys in two-week bursts. Daily FI was measured with 4 yes/no items ranging in severity: worry about food, parent eating less than they should, child eating less than they should, and skipping meals. Items were analyzed as a sum score and individually (to examine FI severity). Among parents who reported FI at least once, FI meaningfully varied day-to-day within individuals (~26% of variation). Different indicators of FI, however, had different proportions of between- and within-person variability: Worry about food, a less severe aspect of FI, had 32% daily variation, whereas the more severe aspect of meal skipping had 45% daily variation. Thus, although substantial between-person differences in FI exist, there is meaningful within-person variability in FI. -person FI variability may be related to the indicator (e.g., severe FI shows greater within-person variability). Considering within-person FI variability, and not just average FI level, may help us understand how FI undermines functioning and how and when best to intervene.

## Introduction

Food insecurity, or lack of consistent access to enough food to live an active and healthy life, is linked with negative health and psychological outcomes across the lifespan [1, 2]. Food insecurity is a widespread issue, but households with children are particularly at risk. Not only are they more likely to experience food insecurity than households without children [3], but its negative effects carry the potential to undermine development of the child(ren) in every domain [2].

Traditionally, food insecurity is measured by asking about experiences with food over the past 12 months, an approach that has led to a better understanding of food insecurity prevalence and informed food assistance policies. There is, however, little understanding of how food insecurity operates across shorter timelines (e.g., weeks or days); as such, a 12-month measure of food insecurity may not accurately capture the everyday experience of food insecurity. Understanding if and how food insecurity varies across shorter time intervals may provide insight into how food insecurity works to disrupt family functioning and child development, something that, despite decades of research on food insecurity prevalence, is still largely not understood. The first step in studying food insecurity across shorter intervals (e.g. days or weeks) is to identify if food insecurity varies on those time scales; this paper addresses this issue by using a daily measure of food insecurity to characterize food insecurity variability and identify if food insecurity is variable across shorter time intervals.

### Food insecurity and children

For children, food insecurity is linked with worse overall health across childhood and increased risk of specific health conditions like anemia and asthma [4, 5]. In addition to undermining physical health, among school-aged children, food insecurity is linked with increased externalizing and internalizing behavior problems [6, 7], and worse math and reading skills [8, 9]. Researchers hypothesize that food insecurity disrupts child development in two primary ways, through inadequate nutrition and stress, both of which are supported throughout the literature. Inadequate nutrition, especially during key developmental windows, may impact functioning thus altering physical and cognitive development [10, 11]. Food insecurity likely works through stress to undermine development by first increasing emotional distress in parents, interfering with their parenting, which in turn, affects their children [2, 12].

### Food insecurity measurement

Food insecurity has historically been conceptualized as something occurring over long periods of time. The primary way food insecurity is measured in the United States is using the U.S. Household Food Security Survey Module [13]. Created by the USDA, the HFSSM askes a variety of questions about experiences with food over the past year, including questions about eating patterns and having enough money to purchase food, but also about the worry that food will run out without enough money to buy more. Using this measure, the USDA estimates that more than 13 million children in the United States were exposed to food insecurity in 2022 [3]. This measure has transformed our understanding of food insecurity. It has documented food insecurity prevalence, identified those at risk, and shaped and informed food assistance programs.

The majority of studies examining the effects of food insecurity and its possible mechanisms use variations of the HFSSM and examine food experiences over the past 12 months to draw cross-sectional or longitudinal conclusions. There is, however, a growing interest and awareness that food insecurity may also vary across shorter periods of time. For example, the Census Pulse tracks food insufficiency using a 7-day reference period [14], and a small body of work uses daily measures of food insecurity [15–19]. Food insecurity encompasses things like hunger, skipping meals, and worry about food, which likely vary over shorter periods of time (e.g., some days will have more worry about food than others).

### Daily food insecurity

To start, we know that food insecurity is not a static construct. Even when it is examined on a yearly level, food insecurity is dynamic, with families transitioning into and out of it [20, 21]. Further, those transitions and patterns into and out of food insecurity are differentially associated with outcomes for children [6, 7]. Early work using daily measures of food insecurity found that food insecurity can vary throughout the SNAP (Supplemental Nutrition Assistance Program) monthly cycle for adults and adolescents. Specifically, food insecurity decreases after the initial SNAP benefit transfer, and increases as benefits decrease throughout the month [16, 22]. Other emerging work using daily measures finds associations between daily food insecurity and outcomes like parent and child well-being and family routines [15, 17]. This initial work documents that there is day-to-day variability in food insecurity, and it has started to highlight the potential in measuring food insecurity using shorter measurement windows. Much about how food insecurity operates on shorter time intervals, however, is unknown. The aim of this paper is to characterize food insecurity within persons, across short time scales. Best practices for daily measurement, and how daily food insecurity measurement could be used moving forward will be discussed.

### The present study

Research using 12-month HFSSM developed a foundational understanding of food insecurity, but we propose that there is value in supplementing this person-level characterization of food insecurity with more (temporally) granular measures that can capture the dynamics of someone’s lived experience. Understanding if and how food insecurity varies across shorter intervals, such as days or weeks, may increase our understanding of this economic stressor in multiple ways. Theoretically, it may provide new insight into the processes through which the long documented negative effects of food insecurity develop. Practically, understanding the timing and patterns of food insecurity across shorter time intervals may inform how and when to best intervene. Variability likely exists on multiple time scales. Research on the SNAP cycles suggest food insecurity varies periodically, within a month [16], but it may also vary day-to-day or week-to-week. For example, if food insecurity varies more over weeks compared to days, it may be that cyclical patterns like paydays or monthly benefit cycles influence much of the variation in food insecurity. Most broadly, it is unlikely that there is one time scale that can adequately characterize all of the nuance in experiences of food insecurity, and exploring if and how food insecurity varies across shorter time scales will provide insight into the lived experience of food insecurity. Using *daily* data on food insecurity among a low-income sample with school-aged children, this study seeks to characterize food insecurity by partitioning its variance into between- and within-person differences to determine the value in considering food insecurity measurement at shorter time intervals.

Partitioning variance in food insecurity into between- and within-person components will contribute to a working understanding of how food insecurity operates on shorter time intervals and provide insight into how best to use a daily measure of food insecurity. In broad terms, between-person variance represents largely stable differences between people, and within-person variance represents fluctuations in food insecurity experienced by a given person over time. The traditional 12-month measure, the HFSSM, largely captures and reflects those stable differences between people, or between-person variance. Using the HFSSM, each individual gets a value and corresponding food insecurity severity category that represents their experience over the past year. By design, the HFSSM does not capture any within-person variance in food insecurity, or how food insecurity may fluctuate within a person. Therefore, when using a daily measure, any variance attributable to within-person differences is potentially unique information not captured by the 12-month measure. Using a measure of daily food insecurity, comparing proportions of between- and within-person variance will help us understand the degree to which food insecurity itself is dynamic, or stable, across short time periods, as well as provide insight into what more frequent (e.g., daily) food insecurity measures may capture to supplement what is obtained using traditional food insecurity measures.

Additionally, food insecurity encompasses many experiences, ranging from worry about food to skipping meals. Although typically considered in aggregate (which is sensible to determine a person-level risk estimate), it is plausible that these experiences are distinct. For example, they reflect different levels of food insecurity severity and may vary differently over time. Thus, we believe it helpful to examine how distinct elements of food insecurity vary within people over short periods of time. This characterization will provide important insight into how food insecurity, and its encompassing experiences, may change among individuals and families over time, across circumstances and events.

## Methods

### Data

Data for this study are drawn from a larger evaluation of a school-based food assistance program, Power Packs Project (PPP). PPP is active throughout southeastern Pennsylvania, but data collection for this study was limited to two counties. After IRB approval, parents were recruited to participate in this study when they signed up for PPP between October and December of 2019. All children who received free and reduced priced lunch were eligible to enroll in PPP. A total of 271 families across six schools signed the consent form to participate in the study. One primary caregiver per household signed up to receive daily text message surveys to their phone (90% mothers). The youngest child in elementary school in the household was selected as the focal child for child focused items, resulting in a sample of children aged 4–11 years old (pre-Kindergarten through 5^th^ grade).

Participants responded via daily text-message surveys for two consecutive weeks each month between January 9^th^ and May 30, 2020. The sample was randomly divided in half, such that half of the sample was being surveyed at a time. One group started on January 9^th^, completed two-weeks of daily surveys, and then the other group completed two weeks of daily surveys. This pattern of two-week measurement bursts followed by two weeks with no surveys continued for five burst cycles, through May 30, 2020. Moving forward we will refer to each two-week period of daily surveys as a “burst”.

### Sample

The families participating in the study were from six Title 1 elementary schools in two school districts in Pennsylvania. This low-income, partially- rural sample was majority Latinx and participated in at least one food assistance program (SNAP or WIC). Demographic information for the sample was collected in a one-time survey distributed in June 2020. If a participant did not complete that survey, their race and education status were drawn from administrative data collected by the PPP team. See Table 1 for full demographic information for the analytic sample.

**Table 1:** Analytic sample demographics.

	Mean or Percentage
Parent education	
Less than HS	23.68%
HS or GED only	42.76%
More than HS	33.55%
Parent race or ethnicity	
Latinx	60.13%
Black non latinx	9.80%
White non Latinx, Mixed Race, Other	30.07%
Survey administered in Spanish	31.37%
Received free/reduced price lunch	90.58%
Received SNAP	63.38%
Received WIC	28.26%
Respondent is child’s mother	89.86%
Respondent average age (SD)	35.07(9.52)
Child grade	
Pre-Kindergarten	21.74%
Kindergarten	13.77%
1st	18.12%
2nd	15.22%
3rd	12.32%
4th	7.97%
5th	10.87%
Number of children in household (SD)	2.49(1.22)
Number of adults in household (SD)	1.96(0.96)
Percent surveys completed (SD)	89.78(13.53)

Abbreviations: GED, general education degree; SNAP, Supplemental Nutrition Assistance Program; WIC, Supplemental Nutrition Assistance Program for Women Infants & Children; SD, standard deviation

Demographics reflect those included in the analytic sample: participants that answered at least 50% of the daily surveys and reported endorsed at least one food insecurity item (mean of food insecurity sum score not equal to 0)

Ns range from 138–153

### Measures

Food insecurity was measured using four yes/no items adapted from the HFSSM [23] to asses daily food insecurity. Child focused items included the name of one focal child, the youngest child in elementary school in the household.

1. Today, were you worried that your food would run out before you got money to buy more?
2. Today, did you eat less than you felt you should because there wasn’t enough money to buy food?
3. Did [child name] eat less today than you felt he/she should because there wasn’t enough money to buy food?
4. Did you or [child name] skip a meal today because your family didn’t have enough money for food?

The first item taps the parent psychological experience of food insecurity, whereas the next three items focus on food insufficiency of both parent and child. Following the approach taken in the HFSSM, responses to the food insecurity items were summed. Items were summed each day to create a daily food insecurity composite score, with a 0–4 range. Responding “no” to each questions yields a score of 0, while answering “yes” to each question results in a score of 4.

### Analytic strategy

To characterize daily food insecurity, we completed a series of null, or empty, multilevel models in order to partition variance and calculate intraclass correlation coefficients (ICCs). We estimated three level models to account for the nested nature of the data, where daily responses (Level 1), were nested within measurement bursts (Level 2), and between different persons (Level 3).

*Total* within-person variance is the sum of variance in daily responses (Level 1) and variance at the measurement burst level (Level 2). Day-to-day variance captures variance within individuals within bursts (and error), whereas burst level variance captures variance within individuals across bursts. Level 3, or person level variance, represents between-person variance, or that which is attributable to largely stable differences between individuals. ICCs were calculated as the proportion of variance of interest to the total variance in the model (See Eqs 1–4). The models and ICCs were estimated and calculated separately for the daily food insecurity sum score (0–4), and each individual food insecurity item.


Within-personvariance=dayvariance+burstvariance (1)

$$Person-level\;ICC=\frac{variance\;of\;random\;intercept}{variance\;of\;random\;intercept+within-subject\;variance}$$ (2)

$$\;Burst-level\;ICC=\frac{burst\;variance}{variance\;of\;random\;intercept+within-subject\;variance}$$ (3)

$$Day-level\;ICC=\frac{day\;variance}{variance\;of\;random\;intercept+within-subject\;variance}$$ (4)

We estimated linear equations and calculated the variance components following the same procedure for the food insecurity sum score and individual food insecurity items. Although the individual food insecurity items were binary (yes/no response options), this approach is appropriate when proportions are not extreme [24].

Given that we are examining variation in daily food insecurity, we limited the analytic sample to only those who reported food insecurity at some point during data collection (resulting in the loss of 27 cases). Meaning, if a participant answered “No” to all four food insecurity items for the entirety of the study period, they were excluded from analyses. Although the entire sample was at risk for food insecurity, if a parent *never* endorsed any food insecurity item in the study period, their responses would artificially lower estimates of variance for food insecurity (as they did not report the experience of food insecurity). As our goal is to partition variance in food insecurity, we limited the sample to those who reported food insecurity at some point during the study period. Further, we limited the sample to those participants who completed at least 50% of the daily surveys to ensure those included in analyses had adequate responses for within-person analyses, resulting in an analytic sample of 153 participants. No significant differences in demographics or food insecurity were present between the analytic and full sample. Overall, participants included in the analytic sample completed 89.78% of the daily surveys, and response rates stayed fairly consistent across the measurement period. The percentage of surveys completed in each of the five 14-day measurement bursts ranged from 86.33% in burst 1 to 87.91% in burst 5, with a peak of 91.97% in burst 4.

## Results

**Fig 1:**
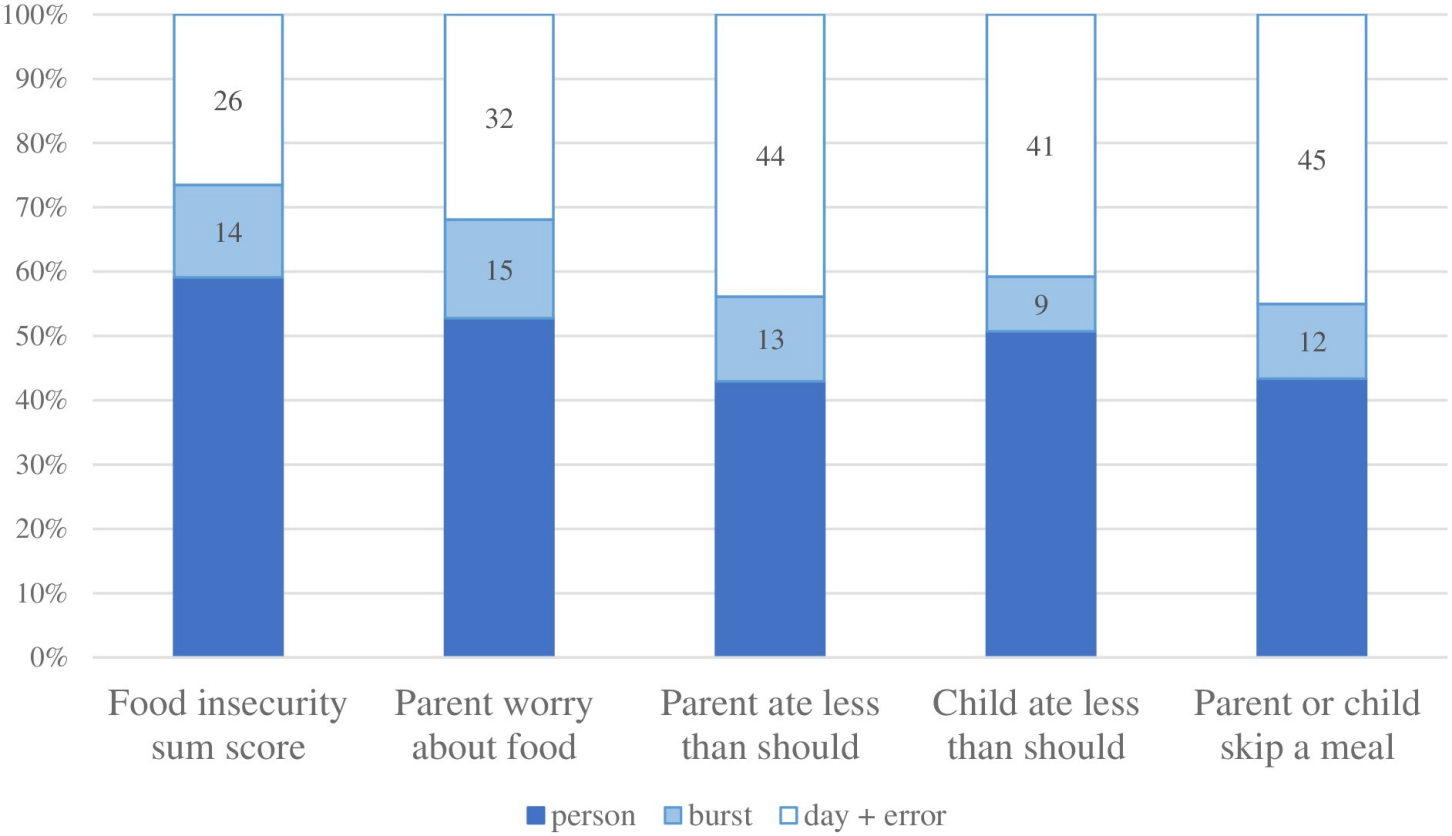
Daily food insecurity: Variance components. Total within-person variance = burst-level variance + day-level variance.

Fig 1 and Table 2 include results from the variance partitioning analyses for the food insecurity sum score and the individual food insecurity items. Analyses reveal that there is variation in food insecurity both between people and within people (variance attributable to both day and burst). ICCs indicate that 59% of the variation in the food insecurity sum score is at the person level, or between persons, with the remaining 40% due to within-person variability (variance attributable to burst, day, and error). The food insecurity sum score showed slightly lower within-person variability compared to the individual food insecurity items, which had proportions of within-person variance ranging from 47%–57%.

**Table 2:** Food insecurity: Intercepts, variances, and variance components.

	Intercept	Level-3 variance	Level-2 variance	Level-1 variance	Person ICC	Burst ICC	Daily ICC (+error)
Food insecurity sum score	0.83 (0.08)	0.84 (0.10)	0.20 (0.01)	0.38 (0.01)	0.59	0.14	0.26
Parent worry about food	0.39 (0.03)	0.12 (0.02)	0.04 (0.002)	0.08 (0.001)	0.53	0.15	0.32
Parent ate less than they should	0.23 (0.02)	0.07 (0.01)	0.02 (0.002)	0.08 (0.001)	0.43	0.13	0.44
Child ate less than they should	0.10 (0.02)	0.05 (0.005)	0.01 (0.001)	0.04 (0.001)	0.51	0.09	0.41
Parent or child skipped a meal	0.12 (0.02)	0.05 (0.01)	0.01 (0.001)	0.05 (0.001)	0.43	0.12	0.45

Notes. Standard errors in parentheses, number of observations range 9513–9568; number of individuals = 153; sample limited to participants who reported experiencing food insecurity and those who answered at least 50% of the daily surveys; proportion of variance attributable to between person differences = Person ICC; proportion of variance attributable to within-person differences = Burst ICC + Daily ICC

A larger proportion of the within-person variability was due to day-to-day variation in responses compared to burst level variability. The proportion of variability attributable to measurement burst was fairly similar across measures, ranging from 9% for children eating less than they should, to 15% for parents worrying food would run out.

### Exploratory analyses

We completed additional analyses to examine if variance components changed over time. Notably, the onset of the COVID-19 pandemic occurred about halfway through data collection, in March of 2020. Previous work, in this data set and others, document that levels of food insecurity initially increased with the onset of COVID-19 [18, 25], but there were also supplemental support policies introduced in response to COVID-19. If and how such changes were related to within-person variability in food insecurity is unknown.

In order to examine how variance in daily food insecurity changed across the study period, we used a similar approach to our main analyses, computing variance components by burst. This allowed us to examine if and how variance components changed across the study period. In order to do this, we completed empty two-level models, accounting only for nesting of days (Level 1) between persons (Level 2). Accounting for burst level variance was not necessary in these models, as each burst was analyzed separately.

**Fig 2:**
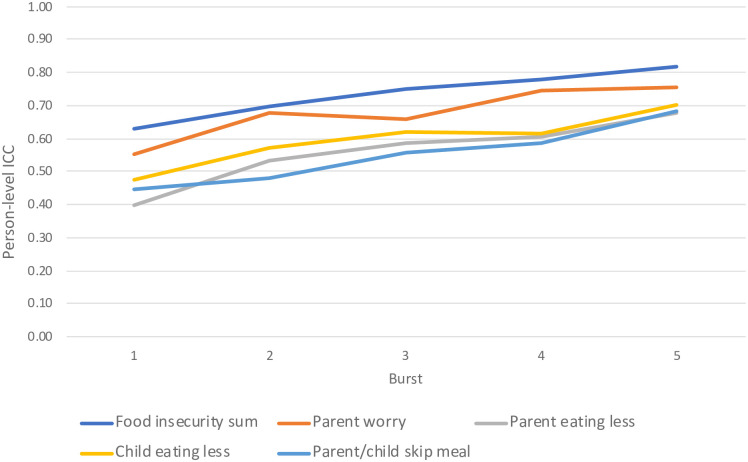
Person level ICCs across study period. This figure represents changes in person-level ICCs across the study period, not means. Person-level ICCs represent between-person variance, or the proportion of variance attributable to differences between persons.

Results from partitioning variance in food insecurity by burst are in Fig 2; these analyses reveal that person-level ICCs (representing between-person variance) for the food insecurity sum score and the individual items increased over time. This indicates that day-to-day variability in food insecurity (not shown in the figure) decreased across the study period. The food insecurity sum score person-level ICC increased by 30% (0.627 to 0.815) while parents eating less than they should had the largest increase of 71% (0.395 to 0.677). By the end of the study period, within-person, or day-to-day, variation was low. Only 18.5% of the variance in the food insecurity sum score was due to day-to-day variability (person-level ICC in burst 5 of 0.815).

We completed further analyses to explore possible reasons for this decrease in within-person variance. First, we examined if food insecurity means decreased over time, as zero-inflated variables should exhibit less variability. Although food insecurity peaked at the onset of COVID-19, mean levels of endorsement at the end of the study period were very similar to those in the beginning of data collection. Therefore, in these data, low endorsement does not appear to explain the decrease in within-person variation.

**Fig 3:**
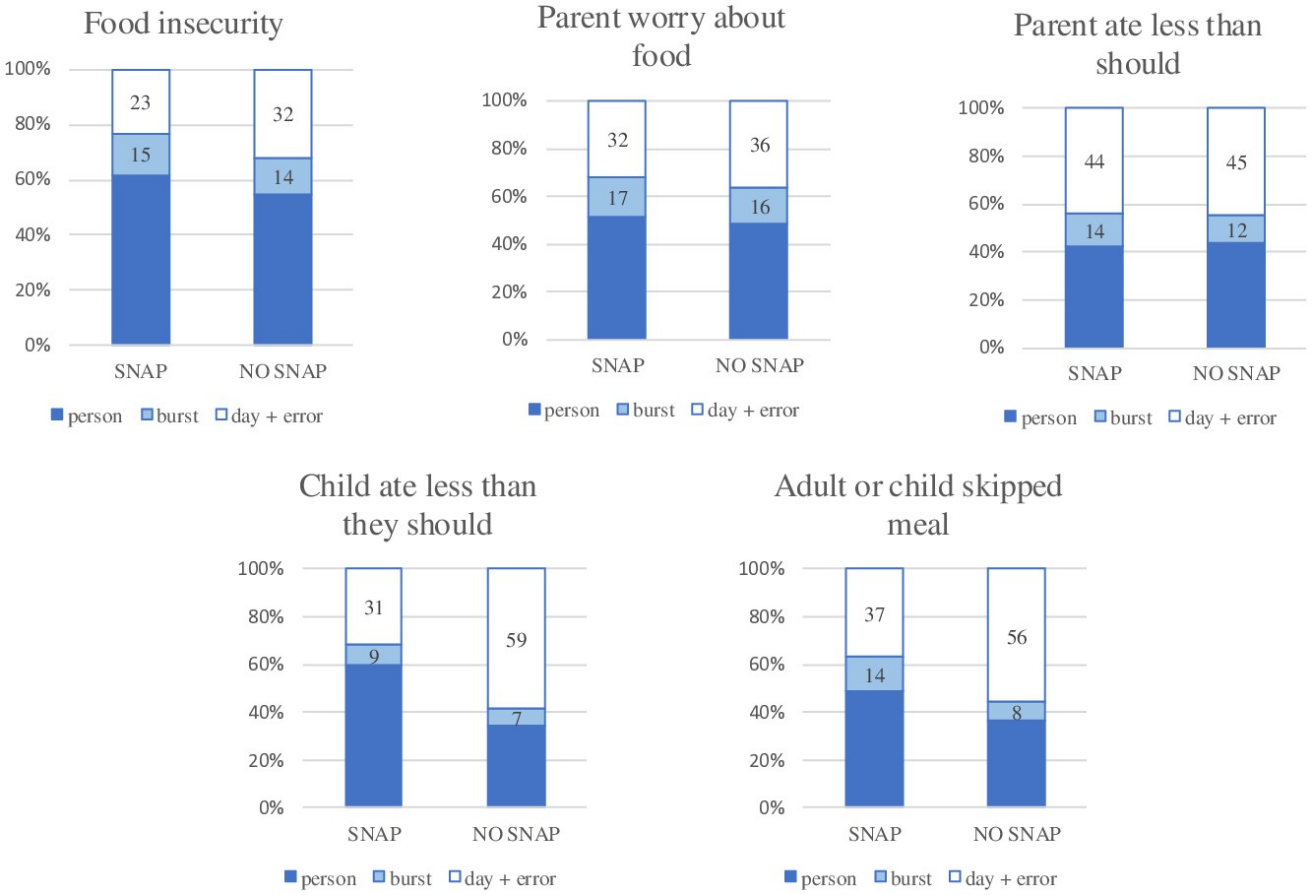
Food insecurity variance components by SNAP status. SNAP, Supplemental Nutrition Assistance Program; SNAP *n* = 90; NO SNAP *n* = 50.

As mentioned, the COVID-19 pandemic and associated school closures began about halfway through the study period, during burst 3. Although rates of food insecurity spiked with the onset of the pandemic, the first programmatic and policy changes were enacted in late March 2020 to help counteract increased food insecurity [26]. It is possible that aid from social programs and policies limited day-to-day variability in food insecurity. To explore this hypothesis, we compared variance components for those participating in SNAP and those not participating in SNAP. We did not have adequate sample size to examine differences in SNAP use before and after the onset of COVID-19, so analyses were conducted with data collapsed across bursts. These exploratory analyses (Fig 3) revealed that the group receiving SNAP had *less* within-person variability, or day-to day variability, than the group who did not receive SNAP. This pattern emerged in the food insecurity sum score, but it was especially pronounced for children eating less than they should and meal skipping. For children eating less than they should, within-person variance accounted for 66% of the variance in the group not receiving SNAP, and only 40% for the group receiving SNAP. For adult or child skipping a meal, within-person variance (burst and day+error) accounted for 64 percent of the variance in the group not receiving SNAP, and only 51 percent of the group receiving SNAP.

## Discussion

Results from this study indicate that food insecurity varies on a daily basis, but that there is heterogeneity in that variation. Overall, about 40% of the variation in food insecurity reflects within-person differences, and a larger proportion of within-person variation across days relative to across bursts demonstrates that it varies more over shorter time scales (days), than longer ones (bursts). Our results are similar to two previous studies that found about 35% of the variance in food insecurity was due to within-person differences, although those studies used slightly different food insecurity measures and different samples [16, 19]. Our analyses divided within-person variance in food insecurity between days and bursts. In these data, measurement bursts represent 14 days of nightly surveys, therefore burst-level variance in food insecurity can be interpreted as more periodic variation, compared to day-to-day. Although the majority of within-person variance of food insecurity is attributable to days, and not bursts, the presence of burst-level variance suggests that some variability in food insecurity may be cyclical in nature. Burst-level variation reflects variation that occurs over a two-week period, which may be associated with cyclical patterns like payroll or SNAP benefit distribution [16].

These results confirm expectations that substantial, largely stable between person differences in food insecurity exist, but extend our understanding to include the view that there is meaningful within-person variability in food insecurity over weeks and days. Additionally, item level analyses reveal differences in variance components by indicator. Indicators of more severe food insecurity (e.g. eating less than you should and skipping meals) show greater within-person variability. Within-person variability was lowest for parent worry about food, the least severe indicator of food insecurity. This was a somewhat surprising finding, as worry is a psychological component of food insecurity, and it seemed plausible to us that such a psychological construct would vary more day-to-day relative to more severe indicators reflective of deprivation (e.g., skipping meals). It could be that this item was worded in such a way that does not capture worry that changes day-to-day, or, it is possible that worry about food is more of a person-level trait (that is, some people may be prone to worry, whereas others are less so).

We should, however, interpret item-level analyses with caution, as any preliminary conclusions are based on single indicators. Further, each item had more within-person variation than the sum score. This may be because individual items contain more measurement error, or it could be because the food insecurity sum score obscures differences between experiences. For example, a score of 2 on the sum score could reflect various experiences. Understanding and exploring these nuances is an important area for future research to identify and optimize which food insecurity items are most suited to within-person measurement.

Next, our exploratory analyses suggest that within-person variability may change in different contexts or circumstances. Between-person variability increased over the study period. Over time, our participants’ responses became more stable. It may be that changes in environmental circumstances may have resulted in decreases in daily variability. With COVID-19 came economic supports and increases in food assistance, and it is possible that these supports smoothed consumption in a way that decreased instability and variance in food insecurity. Results from the exploratory SNAP analyses are supportive of this theory, as those receiving SNAP had less within-person variation. Future work should further examine the association between food assistance and food insecurity variability, but our exploratory results suggest that those who do not receive SNAP may have increased food insecurity instability.

Daily food insecurity may be a useful tool in exploring food insecurity mechanisms and informing food assistance programs. Research should continue, however, to explore how daily variation in food insecurity varies by food insecurity experience and circumstance in order to identify when it is most useful and appropriate to implement. When there are low levels of within-person variation in any construct, daily measurement, which is costly and time consuming for both participants and researchers, may not be a necessary or appropriate approach [27]. Future research should work to characterize contributors to within-person variability in food insecurity and identify under which circumstances food insecurity variability is highest. Further, we should identify if, and how, that variance, or instability in food insecurity, influences functioning. Typically, instability is considered a negative stressor across domains [28, 29]. Thus, it is possible that it is instability or uncertainty in food insecurity which is especially harmful, not just food insecurity severity or level. Daily measurement is a promising method to explore this.

This work should be interpreted with its limitations in mind. To start, we had a relatively small sample size of parents with young children from a singular geographic location, and results may not generalize to other populations. The fairly homogeneous nature of the sample at this specific time period may have made people appear more similar to one another than during a different time, with a different sample. Further, we limited our analytic sample to those who reported food insecurity, meaning results may not generalize to other low-income samples that do not experience food insecurity. Person-level estimates may be an overestimate, as participants in our sample were regionally close and surveyed at the same point in time, maximizing their similarity. It may be easier to detect daily variation in food insecurity among a larger and more heterogeneous sample in location and circumstance. Additionally, we only used four yes/no food insecurity items, so the granularity of information regarding food insecurity experience is limited (e.g., versus using a continuous measure).

In this study, 40 to 57% of the variance in food insecurity was due to within-person differences, leaving 43–60% of the variance due to differences between people. We suspect the typical 12-month measure of food insecurity likely captures the majority of person-level variance in food insecurity in an effective and efficient way, and it should continue to be used. As we move into attempting to capture experiences and flux in food insecurity on a shorter time scale, however, this work provides preliminary guidance on how those items may be adopted or extended to be sensitive to short-term variation. Researchers should consider integrating daily measurement with larger macro scale measurement, episodically, to increase understanding of specific circumstances. For example, short daily measurement bursts strategically timed within a larger study may provide invaluable insight into specific periods of interests or policy changes.

Although substantial and largely stable between-person differences in food insecurity exist, there is meaningful within-person variability in food insecurity. Moreover, within-person food insecurity variability may be related to the indicator (e.g., indicators of more severe FI show greater within-person variability) and to the context (e.g., receiving government supports to reduce food insecurity or not). Such findings highlight the potential importance of predictability and stability in the food insecurity experience. Considering food insecurity variability, and not just level, may help us understand how food insecurity undermines functioning, and how and when best to intervene. If within-person food insecurity variability itself is related to negative outcomes, efforts to reduce food insecurity instability may be a promising and novel way to combat this widespread public health issue. In sum, continued research using daily measurement of food insecurity is a promising tool to better understanding and working to reduce food insecurity and its harmful effects.
